# Supplementary material for: Approaches and results of intersectoral actions for tuberculosis control in the world: A scoping review
Source: PLoS One. 2025 Jun 26;20(6):e0326784. doi: 10.1371/journal.pone.0326784 (PMC12200668; doi:10.1371/journal.pone.0326784)
Supplement: S1 Search Strategies — The document details the process of electronically capturing studies into databases. (DOCX) [file pone.0326784.s001.docx]

**SEARCH STRATEGIES**

The research question chosen was: What is the scientific evidence on the approaches and results of tuberculosis control actions in the global scenario, focusing on intersectorality?. The search strategies used to extract the studies were then defined according to the PCC mnemonic:

- Problem (P): Tuberculosis

- Concept (C): Intersectorality

- Context (C): National Tuberculosis Control Programs, National Tuberculosis Control Policy and Tuberculosis Control Actions, Public Health.

First phase: The descriptors that composed the PCC mnemonic were selected from descriptors of Medical Subject Heading (MeSH), health sciences descriptors (DeCS) and Embase subject Reading (EMTREE). Uncontrolled terms and singular and plural keywords were also included. The construction of the descriptors and search strategies was supported by a librarian.

Second phase: The search strategies in the databases are applied, performing tests and analysis of the titles and abstracts of the selected studies, as well as the terms of the index used to describe the articles. This process took place from February 10 to 27, 2024, when the search strategies were last applied.

The electronic capture of the studies took place in the PubMed, CINAHL, Web of Science, Embase, Scopus databases, access to these databases occurred through the Periodicals Portal of the Coordination for the Improvement of Higher Education Personnel (CAPES), through access by the Federated Academic Community (CAFe) with the login of the Federal University of Rio Grande do Norte, at the electronic address: https://www-periodicos-capes-gov-br.ezl.periodicos.capes.gov.br/.

LILACS was also included, which was accessed through the Virtual Health Library, at the electronic address <https://lilacs.bvsalud.org>

The search in the grey literature was carried out on the portals:

Brazilian Digital Library of Theses and Dissertations <https://bdtd.ibict.br/vufind/>(No results found).

Open Access Scientific Repository of Portugal (RCAAP) https://www.rcaap.pt/

WHO Global Research <https://www.who.int/publications/i>

To carry out this review, Dart-Europe http://www.dart-europe.eu/basic-search.php and the Electronic Theses Online Service (EThOS) https://ethos.bl.uk/Home.do;jsessionid=D21ECD249926BDE2B0B862D7670ED2E7 were considered, but due to technical problems they are disabled for data collection, from the beginning of the research until the date of 28.06.2024, when the final search of the studies was carried out.

All search strategies were applied and adapted according to the databases, however, strategies #1#2#3 and #1#2 were used for searching the databases as they best suited the object of the study and the following filters were applied: Year, language and free text.

| **Objective** | | To map the approaches and results of intersectoral tuberculosis control actions at the global level, with a focus on intersectorality. | | |
| --- | --- | --- | --- | --- |
|  | | **P** | **C** | **C** |
| **Extraction** | | Tuberculose | Intersetorialidade | Programas Nacionais de Controle da Tuberculose  Politica Nacional de Controle da Tuberculose  Ações de controle da Tuberculose  Saúde Pública |
| **Conversion** | | Tuberculose  Tuberculosis | Colaboração Intersetorial  Colaboración Intersectorial | Assistência Integral à Saúde  Programas Nacionais de Saúde  Estratégias de Saúde Nacionais  Gestão em saúde  Saúde Pública  Atención Integral de Salud  Programas Nacionales de Salud  Estrategias de Salud Nacionales  Gestión en Salud  Salud Pública |
| **Combination** | | TB  “Pneumologia Sanitária”  “Infecção por Mycobacterium tuberculosis”  “Infección por Mycobacterium tuberculosis” | “Ação Integrada de Saúde”  “Ação Intersetorial”  “Ação Multissetorial”  “Ações Integradas de Saúde”  “Cooperação Intersetorial”  “Integração dos Serviços de Saúde”  “Intersetorialidade”  “Rede Intersetorial”  “Redes Intersetoriais”  “Acciones Integradas de Salud”  “Acción Integrada de Salud”  “Acción Intersectorial”  “Acción Multisectorial”  “Cooperación Intersectorial”  “Integración de los Servicios de Salud”  “Intersectorialidad”  “Red Intersectorial”  “Redes Intersectoriales” | Atenção Integral à Saúde  Atendimento Integral à Saúde  Estratégias Nacionais de Saúde Estratégias Sanitárias Nacionais  Saúde coletiva  Estrategias de Salud Nacionales  Atención Integral al Anciano Prestación Positiva de Servicios de Salud  Estrategias Nacionales de Salud Estrategias Sanitarias Nacionales |
| **Construction** | | Tuberculose **OR** Tuberculosis  **OR** TB **OR**  “Pneumologia Sanitária” **OR**  “Infecção por Mycobacterium tuberculosis” **OR**  “Infección por Mycobacterium tuberculosis” | “Colaboração Intersetorial” **OR**  “Colaboración Intersectorial” **OR**  “Ação Integrada de Saúde” **OR**  “Ação Intersetorial” **OR**  “Ação Multissetorial” **OR**  “Ações Integradas de Saúde” **OR**  “Cooperação Intersetorial” **OR**  “Integração dos Serviços de Saúde” **OR**  “Intersetorialidade” **OR**  “Rede Intersetorial” **OR**  “Redes Intersetoriais” **OR**  “Acciones Integradas de Salud” **OR**  “Acción Integrada de Salud” **OR**  “Acción Intersectorial” **OR**  “Acción Multisectorial” **OR**  “Cooperación Intersectorial” **OR**  “Integración de los Servicios de Salud” **OR**  “Intersectorialidad” **OR**  “Red Intersectorial” **OR**  “Redes Intersectoriales” | “Assistência Integral à Saúde”” **OR**  “Programas Nacionais de Saúde” **OR**  “Estratégias de Saúde Nacionais” **OR**  “Gestão em saúde” **OR**  “Saúde Pública” **OR**  “Atención Integral de Salud” **OR**  “Programas Nacionales de Salud” **OR**  “Estrategias de Salud Nacionales” **OR**  “Gestión en Salud” **OR**  “Salud Pública” **OR**  “Atenção Integral à Saúde” **OR**  “Atendimento Integral à Saúde” **OR** “Estratégias Nacionais de Saúde” **OR** “Estratégias Sanitárias Nacionais” **OR**  “Estrategias de Salud Nacionales” **OR**  “Atención Integral al Anciano” **OR** “Prestación Positiva de Servicios de Salud” **OR**  “Estrategias Nacionales de Salud” **OR** “Estrategias Sanitarias Nacionales” |
| **Use In Lilacs** | | | | **Records found** |
| **#1** | tuberculose OR tuberculosis OR “TB” OR “pneumologia sanitária” OR “infecção por mycobacterium tuberculosis” OR “infección por mycobacterium tuberculosis” | | | 5771 |
| **#2** | “colaboração intersetorial” OR “colaboración intersectorial” OR “ação integrada de saúde” OR “ação intersetorial” OR “ação multissetorial” OR “ações integradas de saúde” OR “cooperação intersetorial” OR “integração dos serviços de saúde” OR “intersetorialidade” OR “rede intersetorial” OR “redes intersetoriais” OR “acciones integradas de salud” OR “acción integrada de salud” OR “acción intersectorial” OR “acción multisectorial” OR “cooperación intersectorial” OR “integración de los servicios de salud” OR “intersectorialidad” OR “red intersectorial” OR “redes intersectoriales” | | | 862 |
| **#3** | “assistência integral à saúde” OR “programas nacionais de saúde” OR “estratégias de saúde nacionais” OR “gestão em saúde” OR “saúde pública” OR “atención integral de salud” OR “programas nacionales de salud” OR “estrategias de salud nacionales” OR “gestión en salud” OR “salud pública” OR “atenção integral à saúde” OR “atendimento integral à saúde” OR “estratégias nacionais de saúde” OR “estratégias sanitárias nacionais” OR “estrategias de salud nacionales” OR “atención integral al anciano” OR “prestación positiva de servicios de salud” OR “estrategias nacionales de salud” OR “estrategias sanitarias nacionales” | | | 107 |
| **#1 #2 #3** | Tuberculose OR Tuberculosis OR “TB” OR “Pneumologia Sanitária” OR “Infecção por Mycobacterium tuberculosis” OR “Infección por Mycobacterium tuberculosis” AND “Colaboração Intersetorial” OR “Colaboración Intersectorial” OR “Ação Integrada de Saúde” OR “Ação Intersetorial” OR “Ação Multissetorial” OR “Ações Integradas de Saúde” OR “Cooperação Intersetorial” OR “Integração dos Serviços de Saúde” OR “Intersetorialidade” OR “Rede Intersetorial” OR “Redes Intersetoriais” OR “Acciones Integradas de Salud” OR “Acción Integrada de Salud” OR “Acción Intersectorial” OR “Acción Multisectorial” OR “Cooperación Intersectorial” OR “Integración de los Servicios de Salud” OR “Intersectorialidad” OR “Red Intersectorial” OR “Redes Intersectoriales” AND “Assistência Integral à Saúde” OR “Programas Nacionais de Saúde” OR “Estratégias de Saúde Nacionais” OR “Gestão em saúde” OR “Saúde Pública” OR “Atención Integral de Salud” OR “Programas Nacionales de Salud” OR “Estrategias de Salud Nacionales” OR “Gestión en Salud” OR “Salud Pública” OR “Atenção Integral à Saúde” OR “Atendimento Integral à Saúde” OR “Estratégias Nacionais de Saúde” OR “Estratégias Sanitárias Nacionais” OR “Estrategias de Salud Nacionales” OR “Atención Integral al Anciano” OR “Prestación Positiva de Servicios de Salud” OR “Estrategias Nacionales de Salud” OR “Estrategias Sanitarias Nacionales” | | | 0 |
| **#1 #2** | tuberculose OR tuberculosis OR “TB” OR “pneumologia sanitária” OR “infecção por mycobacterium tuberculosis” OR “infección por mycobacterium tuberculosis” AND “colaboração intersetorial” OR “colaboración intersectorial” OR “ação integrada de saúde” OR “ação intersetorial” OR “ação multissetorial” OR “ações integradas de saúde” OR “cooperação intersetorial” OR “integração dos serviços de saúde” OR “intersetorialidade” OR “rede intersetorial” OR “redes intersetoriais” OR “acciones integradas de salud” OR “acción integrada de salud” OR “acción intersectorial” OR “acción multisectorial” OR “cooperación intersectorial” OR “integración de los servicios de salud” OR “intersectorialidad” OR “red intersectorial” OR “redes intersectoriales” | | | 1 |
| **#1 #3** | Tuberculose OR Tuberculosis OR “TB” OR “Pneumologia Sanitária” OR “Infecção por Mycobacterium tuberculosis” OR “Infección por Mycobacterium tuberculosis” AND “Assistência Integral à Saúde” OR “Programas Nacionais de Saúde” OR “Estratégias de Saúde Nacionais” OR “Gestão em saúde” OR “Saúde Pública” OR “Atención Integral de Salud” OR “Programas Nacionales de Salud” OR “Estrategias de Salud Nacionales” OR “Gestión en Salud” OR “Salud Pública” OR “Atenção Integral à Saúde” OR “Atendimento Integral à Saúde” OR “Estratégias Nacionais de Saúde” OR “Estratégias Sanitárias Nacionais” OR “Estrategias de Salud Nacionales” OR “Atención Integral al Anciano” OR “Prestación Positiva de Servicios de Salud” OR “Estrategias Nacionales de Salud” OR “Estrategias Sanitarias Nacionales” | | | 3 |

| **Objective** | | To map the approaches and results of intersectoral tuberculosis control actions at the global level, with a focus on intersectorality. | | | |
| --- | --- | --- | --- | --- | --- |
|  | | **P** | **C** | **C** | |
| **Extraction** | | Tuberculosis | Intersectorality | National Tuberculosis Control Programs  National Tuberculosis Control Policy  Tuberculosis control actions  Public health | |
| **Conversion** | | Tuberculosis | “Intersectoral Collaboration” | “Comprehensive Health Care”  “National Health Programs”  “Population Health Management”  “Public Health” | |
| **Combination** | | Tuberculoses  “Koch's Disease”  “Koch Disease”  “Mycobacterium tuberculosis Infection”  “Infection, Mycobacterium tuberculosis”  “Infections, Mycobacterium tuberculosis”  “Mycobacterium tuberculosis Infections” | “Collaboration, Intersectoral”  “Collaborations, Intersectoral”  “Intersectoral Collaborations”  “Intersectoral Cooperation”  “Cooperation, Intersectoral” | “Health Care, Comprehensive”  “Comprehensive Healthcare”  “Healthcare, Comprehensive”  “Health Program, National”  “Health Programs, National”  “National Health Program”  “Program, National Health”  “Programs, National Health”  “Health Services, National”  “Health Service, National”  “National Health Service”  “Service, National Health”  “Services, National Health”  “National Health Services”  “Health Management, Population”  “Management, Population Health”  “Community Health” | |
| **Construction** | | Tuberculosis **OR** Tuberculoses **OR** “Kochs Disease” **OR** “Koch's Disease” **OR** “Koch Disease” **OR** “Mycobacterium tuberculosis Infection” **OR** “Infection, Mycobacterium tuberculosis” **OR** “Infections, Mycobacterium tuberculosis” **OR**  “Mycobacterium tuberculosis Infections” | “Intersectoral Collaboration” **OR** “Collaborations, Intersectoral” **OR** “Intersectoral Collaborations” **OR** “Intersectoral Cooperation” **OR**  “Cooperation, Intersectoral” | “Comprehensive Health Care” **OR** “National Health Programs” **OR** “Population Health Management” **OR** “Health Care, Comprehensive” **OR**  “Comprehensive Healthcare” **OR**  “Healthcare, Comprehensive” **OR**  “Health Program, National” **OR**  “Health Programs, National” **OR**  “National Health Program” **OR**  “Program, National Health” **OR**  “Programs, National Health” **OR**  “Health Services, National” **OR**  “Health Service, National” **OR**  “National Health Service” **OR**  “Service, National Health” **OR**  “Services, National Health” **OR**  “National Health Services” **OR** “Public Health” **OR** “Community Health” **OR** “Health Management, Population” **OR** “Management, Population Health” | |
| **Use in MEDLINE** | | | | | **Records found** |
| **#1** | ("tuberculosis"[MeSH Terms] OR "tuberculosis"[All Fields]) OR ("tuberculosis"[MeSH Terms] OR "tuberculosis"[All Fields] OR "tuberculoses"[All Fields]) OR "Kochs Disease"[All Fields] OR "Koch's Disease"[All Fields] OR "Koch Disease"[All Fields] OR "Mycobacterium tuberculosis Infection"[All Fields] OR "Infection, Mycobacterium tuberculosis"[All Fields] OR "Infections, Mycobacterium tuberculosis"[All Fields] OR "Mycobacterium tuberculosis Infections"[All Fields] | | | | 394226 |
| **#2** | "Intersectoral Collaboration"[All Fields] OR "Intersectoral Collaborations"[All Fields] OR "Intersectoral Cooperation"[All Fields] OR "Cooperation, Intersectoral"[All Fields] | | | | 3192 |
| **#3** | "Comprehensive Health Care"[All Fields] OR "National Health Programs"[All Fields] OR "Population Health Management"[All Fields] OR "Health Care, Comprehensive"[All Fields] OR "Comprehensive Healthcare"[All Fields] OR "Healthcare, Comprehensive"[All Fields] OR "Health Program, National"[All Fields] OR "Health Programs, National"[All Fields] OR "National Health Program"[All Fields] OR "Program, National Health"[All Fields] OR "Programs, National Health"[All Fields] OR "Health Services, National"[All Fields] OR "Health Service, National"[All Fields] OR "National Health Service"[All Fields] OR "Service, National Health"[All Fields] OR "Services, National Health"[All Fields] OR "National Health Services"[All Fields] OR "Public Health"[All Fields] OR "Community Health"[All Fields] OR "Health Management, Population"[All Fields] OR "Management, Population Health"[All Fields] | | | | 1853559 |
| **#1 #2 #3** | ((("tuberculosis"[MeSH Terms] OR "tuberculosis"[All Fields]) OR ("tuberculosis"[MeSH Terms] OR "tuberculosis"[All Fields] OR "tuberculoses"[All Fields]) OR "Kochs Disease"[All Fields] OR "Koch's Disease"[All Fields] OR "Koch Disease"[All Fields] OR "Mycobacterium tuberculosis Infection"[All Fields] OR "Infection, Mycobacterium tuberculosis"[All Fields] OR "Infections, Mycobacterium tuberculosis"[All Fields] OR "Mycobacterium tuberculosis Infections"[All Fields]) AND ("Intersectoral Collaboration"[All Fields] OR "Intersectoral Collaborations"[All Fields] OR "Intersectoral Cooperation"[All Fields] OR "Cooperation, Intersectoral"[All Fields])) AND ("Comprehensive Health Care"[All Fields] OR "National Health Programs"[All Fields] OR "Population Health Management"[All Fields] OR "Health Care, Comprehensive"[All Fields] OR "Comprehensive Healthcare"[All Fields] OR "Healthcare, Comprehensive"[All Fields] OR "Health Program, National"[All Fields] OR "Health Programs, National"[All Fields] OR "National Health Program"[All Fields] OR "Program, National Health"[All Fields] OR "Programs, National Health"[All Fields] OR "Health Services, National"[All Fields] OR "Health Service, National"[All Fields] OR "National Health Service"[All Fields] OR "Service, National Health"[All Fields] OR "Services, National Health"[All Fields] OR "National Health Services"[All Fields] OR "Public Health"[All Fields] OR "Community Health"[All Fields] OR "Health Management, Population"[All Fields] OR "Management, Population Health"[All Fields]) | | | | 1 |
| **#1 #2** | (("tuberculosis"[MeSH Terms] OR "tuberculosis"[All Fields]) OR ("tuberculosis"[MeSH Terms] OR "tuberculosis"[All Fields] OR "tuberculoses"[All Fields]) OR "Kochs Disease"[All Fields] OR "Koch's Disease"[All Fields] OR "Koch Disease"[All Fields] OR "Mycobacterium tuberculosis Infection"[All Fields] OR "Infection, Mycobacterium tuberculosis"[All Fields] OR "Infections, Mycobacterium tuberculosis"[All Fields] OR "Mycobacterium tuberculosis Infections"[All Fields]) AND ("Intersectoral Collaboration"[All Fields] OR "Intersectoral Collaborations"[All Fields] OR "Intersectoral Cooperation"[All Fields] OR "Cooperation, Intersectoral"[All Fields]) | | | | 449 |
| **#1 #3** | (("tuberculosis"[MeSH Terms] OR "tuberculosis"[All Fields]) OR ("tuberculosis"[MeSH Terms] OR "tuberculosis"[All Fields] OR "tuberculoses"[All Fields]) OR "Kochs Disease"[All Fields] OR "Koch's Disease"[All Fields] OR "Koch Disease"[All Fields] OR "Mycobacterium tuberculosis Infection"[All Fields] OR "Infection, Mycobacterium tuberculosis"[All Fields] OR "Infections, Mycobacterium tuberculosis"[All Fields] OR "Mycobacterium tuberculosis Infections"[All Fields]) AND ("Comprehensive Health Care"[All Fields] OR "National Health Programs"[All Fields] OR "Population Health Management"[All Fields] OR "Health Care, Comprehensive"[All Fields] OR "Comprehensive Healthcare"[All Fields] OR "Healthcare, Comprehensive"[All Fields] OR "Health Program, National"[All Fields] OR "Health Programs, National"[All Fields] OR "National Health Program"[All Fields] OR "Program, National Health"[All Fields] OR "Programs, National Health"[All Fields] OR "Health Services, National"[All Fields] OR "Health Service, National"[All Fields] OR "National Health Service"[All Fields] OR "Service, National Health"[All Fields] OR "Services, National Health"[All Fields] OR "National Health Services"[All Fields] OR "Public Health"[All Fields] OR "Community Health"[All Fields] OR "Health Management, Population"[All Fields] OR "Management, Population Health"[All Fields]) | | | | 16,884 |

| **Objective** | | To map the approaches and results of intersectoral tuberculosis control actions at the global level, with a focus on intersectorality. | | | |
| --- | --- | --- | --- | --- | --- |
|  | | **P** | **C** | **C** | |
| **Extraction** | | Tuberculose | Intersetorialidade | Programas Nacionais de Controle da Tuberculose  Politica Nacional de Controle da Tuberculose  Ações de controle da Tuberculose  Saúde pública | |
| **Conversion** | | Tuberculosis | “Intersectoral Collaboration” | “Comprehensive Health Care”  “National Health Programs”  “Population Health Management”  “Public Health” | |
| **Combination** | | Tuberculoses  “Koch's Disease”  “Koch Disease”  “Mycobacterium tuberculosis Infection”  “Infection, Mycobacterium tuberculosis”  “Infections, Mycobacterium tuberculosis”  “Mycobacterium tuberculosis Infections” | “Collaboration, Intersectoral”  “Collaborations, Intersectoral”  “Intersectoral Collaborations”  “Intersectoral Cooperation”  “Cooperation, Intersectoral” | “Health Care, Comprehensive”  “Comprehensive Healthcare”  “Healthcare, Comprehensive”  “Health Program, National”  “Health Programs, National”  “National Health Program”  “Program, National Health”  “Programs, National Health”  “Health Services, National”  “Health Service, National”  “National Health Service”  “Service, National Health”  “Services, National Health”  “National Health Services”  “Health Management, Population”  “Management, Population Health”  “Community Health” | |
| **Construction** | | Tuberculosis **OR** Tuberculoses **OR** “Kochs Disease” **OR** “Koch's Disease” **OR** “Koch Disease” **OR** “Mycobacterium tuberculosis Infection” **OR** “Infection, Mycobacterium tuberculosis” **OR** “Infections, Mycobacterium tuberculosis” **OR**  “Mycobacterium tuberculosis Infections” | “Intersectoral Collaboration” **OR** “Collaborations, Intersectoral” **OR** “Intersectoral Collaborations” **OR** “Intersectoral Cooperation” **OR**  “Cooperation, Intersectoral” | “Comprehensive Health Care” **OR** “National Health Programs” **OR** “Population Health Management” **OR** “Health Care, Comprehensive” **OR**  “Comprehensive Healthcare” **OR**  “Healthcare, Comprehensive” **OR**  “Health Program, National” **OR**  “Health Programs, National” **OR**  “National Health Program” **OR**  “Program, National Health” **OR**  “Programs, National Health” **OR**  “Health Services, National” **OR**  “Health Service, National” **OR**  “National Health Service” **OR**  “Service, National Health” **OR**  “Services, National Health” **OR**  “National Health Services” **OR** “Public Health” **OR** “Community Health” **OR** “Health Management, Population” **OR** “Management, Population Health” | |
| **Use In CINAHL - EBSCOHOST** | | | | | **Records found** |
| **#1** | Tuberculosis OR Tuberculoses OR “Kochs Disease” OR “Koch's Disease” OR “Koch Disease” OR “Mycobacterium tuberculosis Infection” OR “Infection, Mycobacterium tuberculosis” OR “Infections, Mycobacterium tuberculosis” OR “Mycobacterium tuberculosis Infections” | | | | 456402 |
| **#2** | “Intersectoral Collaboration” OR “Collaborations, Intersectoral” OR “Intersectoral Collaborations” OR “Intersectoral Cooperation” OR “Cooperation, Intersectoral” | | | | 4350 |
| **#3** | “Comprehensive Health Care” OR “National Health Programs” OR “Population Health Management” OR “Health Care, Comprehensive” OR “Comprehensive Healthcare” OR “Healthcare, Comprehensive” OR “Health Program, National” OR “Health Programs, National” OR “National Health Program” OR “Program, National Health” OR “Programs, National Health” OR “Health Services, National” OR “Health Service, National” OR “National Health Service” OR “Service, National Health” OR “Services, National Health” OR “National Health Services” OR “Public Health” OR “Community Health” OR “Health Management, Population” OR “Management, Population Health” | | | | 2146469 |
| **#1 #2 #3** | (Tuberculosis OR Tuberculoses OR “Kochs Disease” OR “Koch's Disease” OR “Koch Disease” OR “Mycobacterium tuberculosis Infection” OR “Infection, Mycobacterium tuberculosis” OR “Infections, Mycobacterium tuberculosis” OR “Mycobacterium tuberculosis Infections”) **AND** (“Intersectoral Collaboration” OR “Collaborations, Intersectoral” OR “Intersectoral Collaborations” OR “Intersectoral Cooperation” OR “Cooperation, Intersectoral”) **AND** (“Comprehensive Health Care” OR “National Health Programs” OR “Population Health Management” OR “Health Care, Comprehensive” OR “Comprehensive Healthcare” OR “Healthcare, Comprehensive” OR “Health Program, National” OR “Health Programs, National” OR “National Health Program” OR “Program, National Health” OR “Programs, National Health” OR “Health Services, National” OR “Health Service, National” OR “National Health Service” OR “Service, National Health” OR “Services, National Health” OR “National Health Services” OR “Public Health” OR “Community Health” OR “Health Management, Population” OR “Management, Population Health”) | | | | 25 |
| **#1 #2** | (Tuberculosis OR Tuberculoses OR “Kochs Disease” OR “Koch's Disease” OR “Koch Disease” OR “Mycobacterium tuberculosis Infection” OR “Infection, Mycobacterium tuberculosis” OR “Infections, Mycobacterium tuberculosis” OR “Mycobacterium tuberculosis Infections”) **AND** (“Intersectoral Collaboration” OR “Collaborations, Intersectoral” OR “Intersectoral Collaborations” OR “Intersectoral Cooperation” OR “Cooperation, Intersectoral”) | | | | 40 |
| **#1 #3** | (Tuberculosis OR Tuberculoses OR “Kochs Disease” OR “Koch's Disease” OR “Koch Disease” OR “Mycobacterium tuberculosis Infection” OR “Infection, Mycobacterium tuberculosis” OR “Infections, Mycobacterium tuberculosis” OR “Mycobacterium tuberculosis Infections”) **AND (**“Comprehensive Health Care” OR “National Health Programs” OR “Population Health Management” OR “Health Care, Comprehensive” OR “Comprehensive Healthcare” OR “Healthcare, Comprehensive” OR “Health Program, National” OR “Health Programs, National” OR “National Health Program” OR “Program, National Health” OR “Programs, National Health” OR “Health Services, National” OR “Health Service, National” OR “National Health Service” OR “Service, National Health” OR “Services, National Health” OR “National Health Services” OR “Public Health” OR “Community Health” OR “Health Management, Population” OR “Management, Population Health”) | | | | 27246 |

| **Objective** | | To map the approaches and results of intersectoral tuberculosis control actions at the global level, with a focus on intersectorality. | | | |
| --- | --- | --- | --- | --- | --- |
|  | | **P** | **C** | **C** | |
| **Extraction** | | Tuberculose | Intersetorialidade | Programas Nacionais de Controle da Tuberculose  Politica Nacional de Controle da Tuberculose  Ações de controle da Tuberculose  Saúde pública | |
| **Conversion** | | Tuberculosis | “Intersectoral Collaboration” | “Comprehensive Health Care”  “National Health Programs”  “Population Health Management”  “Public Health” | |
| **Combination** | | Tuberculoses  “Koch's Disease”  “Koch Disease”  “Mycobacterium tuberculosis Infection”  “Infection, Mycobacterium tuberculosis”  “Infections, Mycobacterium tuberculosis”  “Mycobacterium tuberculosis Infections” | “Collaboration, Intersectoral”  “Collaborations, Intersectoral”  “Intersectoral Collaborations”  “Intersectoral Cooperation”  “Cooperation, Intersectoral” | “Health Care, Comprehensive”  “Comprehensive Healthcare”  “Healthcare, Comprehensive”  “Health Program, National”  “Health Programs, National”  “National Health Program”  “Program, National Health”  “Programs, National Health”  “Health Services, National”  “Health Service, National”  “National Health Service”  “Service, National Health”  “Services, National Health”  “National Health Services”  “Health Management, Population”  “Management, Population Health”  “Community Health” | |
| **Construction** | | Tuberculosis **OR** Tuberculoses **OR** “Kochs Disease” **OR** “Koch's Disease” **OR** “Koch Disease” **OR** “Mycobacterium tuberculosis Infection” **OR** “Infection, Mycobacterium tuberculosis” **OR** “Infections, Mycobacterium tuberculosis” **OR**  “Mycobacterium tuberculosis Infections” | “Intersectoral Collaboration” **OR** “Collaborations, Intersectoral” **OR** “Intersectoral Collaborations” **OR** “Intersectoral Cooperation” **OR**  “Cooperation, Intersectoral” | “Comprehensive Health Care” **OR** “National Health Programs” **OR** “Population Health Management” **OR** “Health Care, Comprehensive” **OR**  “Comprehensive Healthcare” **OR**  “Healthcare, Comprehensive” **OR**  “Health Program, National” **OR**  “Health Programs, National” **OR**  “National Health Program” **OR**  “Program, National Health” **OR**  “Programs, National Health” **OR**  “Health Services, National” **OR**  “Health Service, National” **OR**  “National Health Service” **OR**  “Service, National Health” **OR**  “Services, National Health” **OR**  “National Health Services” **OR** “Public Health” **OR** “Community Health” **OR** “Health Management, Population” **OR** “Management, Population Health” | |
| **Use in Web of Science** | | | | | **Records found** |
| **#1** | Tuberculosis OR Tuberculoses OR “Kochs Disease” OR “Koch's Disease” OR “Koch Disease” OR “Mycobacterium tuberculosis Infection” OR “Infection, Mycobacterium tuberculosis” OR “Infections, Mycobacterium tuberculosis” OR “Mycobacterium tuberculosis Infections” | | | | 16202 |
| **#2** | “Intersectoral Collaboration” OR “Collaborations, Intersectoral” OR “Intersectoral Collaborations” OR “Intersectoral Cooperation” OR “Cooperation, Intersectoral” | | | | 947 |
| **#3** | “Comprehensive Health Care” OR “National Health Programs” OR “Population Health Management” OR “Health Care, Comprehensive” OR “Comprehensive Healthcare” OR “Healthcare, Comprehensive” OR “Health Program, National” OR “Health Programs, National” OR “National Health Program” OR “Program, National Health” OR “Programs, National Health” OR “Health Services, National” OR “Health Service, National” OR “National Health Service” OR “Service, National Health” OR “Services, National Health” OR “National Health Services” OR “Public Health” OR “Community Health” OR “Health Management, Population” OR “Management, Population Health” | | | | 797162 |
| **#1 #2 #3** | (Tuberculosis OR tuberculosis OR “Kochs Disease” OR “Koch's Disease” OR “Koch Disease” OR “Mycobacterium tuberculosis Infection” OR “Infection, Mycobacterium tuberculosis” OR “Infections, Mycobacterium tuberculosis” OR “Mycobacterium tuberculosis Infections”) AND (“Intersectoral Collaboration” OR “Collaborations, Intersectoral” OR “Intersectoral Collaborations” OR “Intersectoral Cooperation” OR “Cooperation, Intersectoral”) AND (“Comprehensive Health Care” OR “National Health Programs” OR “Population Health Management” OR “Health Care, Comprehensive” OR “Comprehensive Healthcare” OR “Healthcare, Comprehensive” OR “Health Program, National” OR “Health Programs, National” OR “National Health Program” OR “Program, National Health” OR “Programs, National Health” OR “Health Services, National” OR “Health Service, National” OR “National Health Service” OR “Service, National Health” OR “Services, National Health” OR “National Health Services” OR “Public Health” OR “Community Health” OR “Health Management, Population” OR “Management, Population Health”) | | | | :04 |
| **#1 #2** | (Tuberculosis OR tuberculosis OR “Kochs Disease” OR “Koch's Disease” OR “Koch Disease” OR “Mycobacterium tuberculosis Infection” OR “Infection, Mycobacterium tuberculosis” OR “Infections, Mycobacterium tuberculosis” OR “Mycobacterium tuberculosis Infections”) AND (“Intersectoral Collaboration” OR “Collaborations, Intersectoral” OR “Intersectoral Collaborations” OR “Intersectoral Cooperation” OR “Cooperation, Intersectoral”) | | | | 15 |
| **#1 #3** | (Tuberculosis OR Tuberculoses OR “Kochs Disease” OR “Koch's Disease” OR “Koch Disease” OR “Mycobacterium tuberculosis Infection” OR “Infection, Mycobacterium tuberculosis” OR “Infections, Mycobacterium tuberculosis” OR “Mycobacterium tuberculosis Infections”) **AND** (“Comprehensive Health Care” OR “National Health Programs” OR “Population Health Management” OR “Health Care, Comprehensive” OR “Comprehensive Healthcare” OR “Healthcare, Comprehensive” OR “Health Program, National” OR “Health Programs, National” OR “National Health Program” OR “Program, National Health” OR “Programs, National Health” OR “Health Services, National” OR “Health Service, National” OR “National Health Service” OR “Service, National Health” OR “Services, National Health” OR “National Health Services” OR “Public Health” OR “Community Health” OR “Health Management, Population” OR “Management, Population Health”) | | | | 16202 |

| **Objective** | | To map the approaches and results of intersectoral tuberculosis control actions at the global level, with a focus on intersectorality. | | | |
| --- | --- | --- | --- | --- | --- |
|  | | **P** | **C** | **C** | |
| **Extraction** | | Tuberculose | Intersetorialidade | Programas Nacionais de Controle da Tuberculose  Politica Nacional de Controle da Tuberculose  Ações de controle da Tuberculose  Saúde pública | |
| **Conversion** | | Tuberculosis | “Intersectoral Collaboration” | “Comprehensive Health Care”  “National Health Programs”  “Population Health Management”  “Public Health” | |
| **Combination** | | Tuberculoses  “Koch's Disease”  “Koch Disease”  “Mycobacterium tuberculosis Infection”  “Infection, Mycobacterium tuberculosis”  “Infections, Mycobacterium tuberculosis”  “Mycobacterium tuberculosis Infections” | “Collaboration, Intersectoral”  “Collaborations, Intersectoral”  “Intersectoral Collaborations”  “Intersectoral Cooperation”  “Cooperation, Intersectoral” | “Health Care, Comprehensive”  “Comprehensive Healthcare”  “Healthcare, Comprehensive”  “Health Program, National”  “Health Programs, National”  “National Health Program”  “Program, National Health”  “Programs, National Health”  “Health Services, National”  “Health Service, National”  “National Health Service”  “Service, National Health”  “Services, National Health”  “National Health Services”  “Health Management, Population”  “Management, Population Health”  “Community Health” | |
| **Construction** | | Tuberculosis **OR** Tuberculoses **OR** “Kochs Disease” **OR** “Koch's Disease” **OR** “Koch Disease” **OR** “Mycobacterium tuberculosis Infection” **OR** “Infection, Mycobacterium tuberculosis” **OR** “Infections, Mycobacterium tuberculosis” **OR**  “Mycobacterium tuberculosis Infections” | “Intersectoral Collaboration” **OR** “Collaborations, Intersectoral” **OR** “Intersectoral Collaborations” **OR** “Intersectoral Cooperation” **OR**  “Cooperation, Intersectoral” | “Comprehensive Health Care” **OR** “National Health Programs” **OR** “Population Health Management” **OR** “Health Care, Comprehensive” **OR**  “Comprehensive Healthcare” **OR**  “Healthcare, Comprehensive” **OR**  “Health Program, National” **OR**  “Health Programs, National” **OR**  “National Health Program” **OR**  “Program, National Health” **OR**  “Programs, National Health” **OR**  “Health Services, National” **OR**  “Health Service, National” **OR**  “National Health Service” **OR**  “Service, National Health” **OR**  “Services, National Health” **OR**  “National Health Services” **OR** “Public Health” **OR** “Community Health” **OR** “Health Management, Population” **OR** “Management, Population Health” | |
| **Use in Scopus** | | | | | **Records found** |
| **#1** | tuberculosis OR tuberculoses OR "Kochs Disease" OR "Koch's Disease" OR "Koch Disease" OR "Mycobacterium tuberculosis Infection" OR "Infection, Mycobacterium tuberculosis" OR "Infections, Mycobacterium tuberculosis" OR "Mycobacterium tuberculosis Infections" | | | | 368162 |
| **#2** | "Intersectoral Collaboration" OR "Collaborations, Intersectoral" OR "Intersectoral Collaborations" OR "Intersectoral Cooperation" OR "Cooperation, Intersectoral" | | | | 6693 |
| **#3** | "Comprehensive Health Care" OR "National Health Programs" OR "Population Health Management" OR "Health Care, Comprehensive" OR "Comprehensive Healthcare" OR "Healthcare, Comprehensive" OR "Health Program, National" OR "Health Programs, National" OR "National Health Program" OR "Program, National Health" OR "Programs, National Health" OR "Health Services, National" OR "Health Service, National" OR "National Health Service" OR "Service, National Health" OR "Services, National Health" OR "National Health Services" OR "Public Health" OR "Community Health" OR "Health Management, Population" OR "Management, Population Health" | | | | 909256 |
| **#1 #2 #3** | (Tuberculosis OR Tuberculoses OR “Kochs Disease” OR “Koch's Disease” OR “Koch Disease” OR “Mycobacterium tuberculosis Infection” OR “Infection, Mycobacterium tuberculosis” OR “Infections, Mycobacterium tuberculosis” OR “Mycobacterium tuberculosis Infections”) **AND** (“Intersectoral Collaboration” OR “Collaborations, Intersectoral” OR “Intersectoral Collaborations” OR “Intersectoral Cooperation” OR “Cooperation, Intersectoral”) **AND** (“Comprehensive Health Care” OR “National Health Programs” OR “Population Health Management” OR “Health Care, Comprehensive” OR “Comprehensive Healthcare” OR “Healthcare, Comprehensive” OR “Health Program, National” OR “Health Programs, National” OR “National Health Program” OR “Program, National Health” OR “Programs, National Health” OR “Health Services, National” OR “Health Service, National” OR “National Health Service” OR “Service, National Health” OR “Services, National Health” OR “National Health Services” OR “Public Health” OR “Community Health” OR “Health Management, Population” OR “Management, Population Health”) | | | | 26 |
| **#1 #2** | (Tuberculosis OR Tuberculoses OR “Kochs Disease” OR “Koch's Disease” OR “Koch Disease” OR “Mycobacterium tuberculosis Infection” OR “Infection, Mycobacterium tuberculosis” OR “Infections, Mycobacterium tuberculosis” OR “Mycobacterium tuberculosis Infections”) **AND** (“Intersectoral TTTCollaboration” OR “Collaborations, Intersectoral” OR “Intersectoral Collaborations” OR “Intersectoral Cooperation” OR “Cooperation, Intersectoral”) | | | | 74 |
| **#1 #3** | (Tuberculosis OR Tuberculoses OR “Kochs Disease” OR “Koch's Disease” OR “Koch Disease” OR “Mycobacterium tuberculosis Infection” OR “Infection, Mycobacterium tuberculosis” OR “Infections, Mycobacterium tuberculosis” OR “Mycobacterium tuberculosis Infections”) **AND** (“Comprehensive Health Care” OR “National Health Programs” OR “Population Health Management” OR “Health Care, Comprehensive” OR “Comprehensive Healthcare” OR “Healthcare, Comprehensive” OR “Health Program, National” OR “Health Programs, National” OR “National Health Program” OR “Program, National Health” OR “Programs, National Health” OR “Health Services, National” OR “Health Service, National” OR “National Health Service” OR “Service, National Health” OR “Services, National Health” OR “National Health Services” OR “Public Health” OR “Community Health” OR “Health Management, Population” OR “Management, Population Health”) | | | | 18908 |

| **Objective** | | To map the approaches and results of intersectoral tuberculosis control actions at the global level, with a focus on intersectorality. | | | |
| --- | --- | --- | --- | --- | --- |
|  | | **P** | **C** | **C** | |
| **Extraction** | | Tuberculosis | Intersectorality | National Tuberculosis Control Programs  National Tuberculosis Control Policy  Tuberculosis control actions  Public health | |
| **Conversion** | | Tuberculosis | Intersectoral Collaboration  Collaborative Care Team  Multidisciplinary Team | Health program  Health care management  Health care policy  National health organization  Public Health  Population health management | |
| **Combination** | | 'active TB'  'active tuberculosis'  'case of TB'  'cases of TB'  'chronic tuberculosis'  'infection by M. tuberculosis'  'infection by Mycobacterium tuberculosis'  'infection due to M. tuberculosis'  'infection due to Mycobacterium tuberculosis'  'infection of M. tuberculosis'  'infection of Mycobacterium tuberculosis'  'Koch`s disease'  'M. tuberculosis infection' 'minimal tuberculosis'  'minimum tuberculosis'  'Mycobacterium tuberculosis infection'  'TB (tuberculosis)'  'TB case'  'TB cases'  'TB disease'  'TB infection'  'tuberculous infection'  'tuberculous lesion'  'tuberculosis' | 'Intersectoral Collaboration'  'Collaborative Care Team'  'Multidisciplinary Team'  'collaborative health care team' 'collaborative healthcare team' 'collaborative patient care team' 'inter-disciplinary care team' 'interdisciplinary care team' 'interdisciplinary patient care team'  'multi-disciplinary care team' 'multidisciplinary care team' 'multidisciplinary patient care team'  'collaborative care team' 'inter-disciplinary team'  'interdisciplinary team'  'multi-disciplinary team' | 'Comprehensive health care'  'health care program'  'health care programme'  'health programme'  'healthcare program'  'healthcare programme'  'program, health'  'programme, health'  'health program'  'patient protection and affordable care act'  'policy, health care'  'health care policy'  'national health organisation' 'national health organization'  'national health'  'national health programmes'  'national health programs'  'national health project'  'population-based  health management'  'population health management' | |
| **Construction** | | Tuberculosis **OR** 'active 'active TB' **OR** active tuberculosis' **OR** 'case of TB' **OR** 'cases of TB' **OR** 'chronic tuberculosis' **OR** 'infection by M. tuberculosis' **OR** 'infection by Mycobacterium tuberculosis' **OR** 'infection due to M. tuberculosis' **OR** 'infection due to Mycobacterium tuberculosis' **OR** 'infection of M. tuberculosis' **OR** 'infection of Mycobacterium tuberculosis' **OR** 'Koch`s disease' **OR** 'M. tuberculosis infection' **OR** 'minimal tuberculosis' **OR** 'minimum tuberculosis' **OR** 'Mycobacterium tuberculosis infection' **OR** 'TB (tuberculosis)' **OR** 'TB case' **OR** 'TB cases' **OR** 'TB disease' **OR** 'TB infection' **OR '**tuberculous infection' **OR** 'tuberculous lesion' **OR** 'tuberculosis' | 'Intersectoral Collaboration' **OR**  'Collaborative Care Team' **OR**  'Multidisciplinary Team' **OR** 'Intersectoral Collaboration' **OR**  'Collaborative Care Team' **OR**  'Multidisciplinary Team' **OR**  'collaborative health care team' **OR** 'collaborative healthcare team' **OR** 'collaborative patient care team' **OR** 'inter-disciplinary care team' **OR** 'interdisciplinary care team' **OR** 'interdisciplinary patient care team' **OR** 'multi-disciplinary care team' **OR** 'multidisciplinary care team' **OR** 'multidisciplinary patient care team' **OR** 'collaborative care team' 'inter-disciplinary team' **OR** 'interdisciplinary team' **OR** 'multi-disciplinary team' | 'Health program' **OR**  'Health care management' **OR**  'Health care policy' **OR**  'National health organization' **OR**  'Public Health' **OR**  'Population health management' **OR**  'Comprehensive health care' **OR**  'health care program' **OR**  'health care programme' **OR**  'health programme' **OR**  'healthcare program' **OR**  'healthcare programme' **OR**  'program, health' **OR**  'programme, health' **OR**  'health program' **OR**  'patient protection and affordable care act' **OR**  'policy, health care' **OR**  'health care policy' **OR**  'national health organisation' **OR**  'national health organization' **OR**  'national health' **OR**  'national health programmes' **OR**  'national health programs' **OR**  'national health project' **OR**  'population-based health management' **OR**  'population health management' | |
| **Use in Embase** | | | | | **Records found** |
| **#1** | 'tuberculosis'/exp or 'active TB' OR 'active tuberculosis' OR 'case of TB' OR 'cases of TB' OR 'chronic tuberculosis' OR 'infection by M. tuberculosis' OR 'infection by Mycobacterium tuberculosis' OR 'infection due to M. tuberculosis' OR 'infection due to Mycobacterium tuberculosis' OR 'infection of M. tuberculosis' OR 'infection of Mycobacterium tuberculosis' OR 'Koch`s disease' OR 'M. tuberculosis infection' OR 'minimal tuberculosis' OR 'minimum tuberculosis' OR 'Mycobacterium tuberculosis infection' OR 'TB (tuberculosis)' OR 'TB case' OR 'TB cases' OR 'TB disease' OR 'TB infection' OR 'tuberculous infection' OR 'tuberculous lesion' OR 'tuberculosis' | | | | 403249 |
| **#2** | 'intersectoral collaboration'/exp OR 'intersectoral collaboration' OR 'collaborative care team'/exp OR 'collaborative care team' OR 'multidisciplinary team'/exp or 'collaborative health care team' OR 'collaborative healthcare team' OR 'collaborative patient care team' OR 'inter-disciplinary care team' OR 'interdisciplinary care team' OR 'interdisciplinary patient care team' OR 'multi-disciplinary care team' OR 'multidisciplinary care team' OR 'multidisciplinary patient care team' OR 'collaborative care team' 'inter-disciplinary team' OR 'interdisciplinary team' OR 'multi-disciplinary team' | | | | 13533 |
| **#3** | 'health care management'/exp OR 'health care management' OR 'public health'/exp OR 'public health' OR 'comprehensive health care'/exp OR 'comprehensive health care' OR 'health care program'/exp OR 'health care program' OR 'health care programme'/exp OR 'health care programme' OR 'health programme'/exp OR 'health programme' OR 'healthcare program'/exp OR 'healthcare program' OR 'healthcare programme'/exp OR 'healthcare programme' OR 'program, health'/exp OR 'program, health' OR 'programme, health'/exp OR 'programme, health' OR 'health program'/exp OR 'health program' OR 'patient protection and affordable care act'/exp OR 'patient protection and affordable care act' OR 'policy, health care'/exp OR 'policy, health care' OR 'health care policy'/exp OR 'health care policy' OR 'national health organisation'/exp OR 'national health organisation' OR 'national health organization'/exp OR 'national health organization' OR 'national health'/exp OR 'national health' OR 'national health programmes'/exp OR 'national health programmes' OR 'national health programs'/exp OR 'national health programs' OR 'national health project'/exp OR 'national health project' OR 'population-based health management'/exp OR 'population-based health management' OR 'population health management'/exp OR 'population health management' | | | | 9215170 |
| **#1 #2 #3** | ('tuberculosis'/exp OR 'active tb' OR 'active tuberculosis' OR 'case of tb' OR 'cases of tb' OR 'chronic tuberculosis' OR 'infection by m. tuberculosis' OR 'infection by mycobacterium tuberculosis' OR 'infection due to m. tuberculosis' OR 'infection due to mycobacterium tuberculosis' OR 'infection of m. tuberculosis' OR 'infection of mycobacterium tuberculosis' OR 'koch`s disease' OR 'm. tuberculosis infection' OR 'minimal tuberculosis' OR 'minimum tuberculosis' OR 'mycobacterium tuberculosis infection' OR 'tb (tuberculosis)' OR 'tb case' OR 'tb cases' OR 'tb disease' OR 'tb infection' OR 'tuberculous infection' OR 'tuberculous lesion' OR 'tuberculosis') AND (('intersectoral collaboration'/exp OR 'intersectoral collaboration' OR 'collaborative care team'/exp OR 'multidisciplinary team'/exp OR 'collaborative health care team' OR 'collaborative healthcare team' OR 'collaborative patient care team' OR 'inter-disciplinary care team' OR 'interdisciplinary care team' OR 'interdisciplinary patient care team' OR 'multi-disciplinary care team' OR 'multidisciplinary care team' OR 'multidisciplinary patient care team' OR 'collaborative care team') AND 'inter-disciplinary team' OR 'interdisciplinary team' OR 'multi-disciplinary team') AND ('health care management'/exp OR 'health care management' OR 'public health'/exp OR 'public health' OR 'comprehensive health care'/exp OR 'comprehensive health care' OR 'health care program'/exp OR 'health care program' OR 'health care programme'/exp OR 'health care programme' OR 'health programme'/exp OR 'health programme' OR 'healthcare program'/exp OR 'healthcare program' OR 'healthcare programme'/exp OR 'healthcare programme' OR 'program, health'/exp OR 'program, health' OR 'programme, health'/exp OR 'programme, health' OR 'health program'/exp OR 'health program' OR 'patient protection and affordable care act'/exp OR 'patient protection and affordable care act' OR 'policy, health care'/exp OR 'policy, health care' OR 'health care policy'/exp OR 'health care policy' OR 'national health organisation'/exp OR 'national health organisation' OR 'national health organization'/exp OR 'national health organization' OR 'national health'/exp OR 'national health' OR 'national health programmes'/exp OR 'national health programmes' OR 'national health programs'/exp OR 'national health programs' OR 'national health project'/exp OR 'national health project' OR 'population-based health management'/exp OR 'population-based health management' OR 'population health management'/exp OR 'population health management') | | | | 22 |
| **#1 #2** | ('tuberculosis'/exp OR 'active tb' OR 'active tuberculosis' OR 'case of tb' OR 'cases of tb' OR 'chronic tuberculosis' OR 'infection by m. tuberculosis' OR 'infection by mycobacterium tuberculosis' OR 'infection due to m. tuberculosis' OR 'infection due to mycobacterium tuberculosis' OR 'infection of m. tuberculosis' OR 'infection of mycobacterium tuberculosis' OR 'koch`s disease' OR 'm. tuberculosis infection' OR 'minimal tuberculosis' OR 'minimum tuberculosis' OR 'mycobacterium tuberculosis infection' OR 'tb (tuberculosis)' OR 'tb case' OR 'tb cases' OR 'tb disease' OR 'tb infection' OR 'tuberculous infection' OR 'tuberculous lesion' OR 'tuberculosis') AND (('intersectoral collaboration'/exp OR 'intersectoral collaboration' OR 'collaborative care team'/exp OR 'multidisciplinary team'/exp OR 'collaborative health care team' OR 'collaborative healthcare team' OR 'collaborative patient care team' OR 'inter-disciplinary care team' OR 'interdisciplinary care team' OR 'interdisciplinary patient care team' OR 'multi-disciplinary care team' OR 'multidisciplinary care team' OR 'multidisciplinary patient care team' OR 'collaborative care team') AND 'inter-disciplinary team' OR 'interdisciplinary team' OR 'multi-disciplinary team') | | | | 38 |
| **#1 #3** | ('tuberculosis'/exp OR 'active tb' OR 'active tuberculosis' OR 'case of tb' OR 'cases of tb' OR 'chronic tuberculosis' OR 'infection by m. tuberculosis' OR 'infection by mycobacterium tuberculosis' OR 'infection due to m. tuberculosis' OR 'infection due to mycobacterium tuberculosis' OR 'infection of m. tuberculosis' OR 'infection of mycobacterium tuberculosis' OR 'koch`s disease' OR 'm. tuberculosis infection' OR 'minimal tuberculosis' OR 'minimum tuberculosis' OR 'mycobacterium tuberculosis infection' OR 'tb (tuberculosis)' OR 'tb case' OR 'tb cases' OR 'tb disease' OR 'tb infection' OR 'tuberculous infection' OR 'tuberculous lesion' OR 'tuberculosis') AND ('health care management'/exp OR 'health care management' OR 'public health'/exp OR 'public health' OR 'comprehensive health care'/exp OR 'comprehensive health care' OR 'health care program'/exp OR 'health care program' OR 'health care programme'/exp OR 'health care programme' OR 'health programme'/exp OR 'health programme' OR 'healthcare program'/exp OR 'healthcare program' OR 'healthcare programme'/exp OR 'healthcare programme' OR 'program, health'/exp OR 'program, health' OR 'programme, health'/exp OR 'programme, health' OR 'health program'/exp OR 'health program' OR 'patient protection and affordable care act'/exp OR 'patient protection and affordable care act' OR 'policy, health care'/exp OR 'policy, health care' OR 'health care policy'/exp OR 'health care policy' OR 'national health organisation'/exp OR 'national health organisation' OR 'national health organization'/exp OR 'national health organization' OR 'national health'/exp OR 'national health' OR 'national health programmes'/exp OR 'national health programmes' OR 'national health programs'/exp OR 'national health programs' OR 'national health project'/exp OR 'national health project' OR 'population-based health management'/exp OR 'population-based health management' OR 'population health management'/exp OR 'population health management') | | | | 103171 |

**Grey Literature**

| **Objective** | | To map the approaches and results of intersectoral tuberculosis control actions at the global level, with a focus on intersectorality. | | | |
| --- | --- | --- | --- | --- | --- |
|  | | **P** | **C** | **C** | |
| **Extraction** | | Tuberculosis | Intersectorality | National Tuberculosis Control Programs  National Tuberculosis Control Policy  Tuberculosis control actions  Public health | |
| **Conversion** | | Tuberculose  Tuberculosis | Colaboração Intersetorial  Colaboración Intersectorial | Assistência Integral à Saúde  Programas Nacionais de Saúde  Estratégias de Saúde Nacionais  Gestão em saúde  Saúde Pública  Atención Integral de Salud  Programas Nacionales de Salud  Estrategias de Salud Nacionales  Gestión en Salud  Salud Pública | |
| **Combination** | | TB  “Pneumologia Sanitária”  “Infecção por Mycobacterium tuberculosis”  “Infección por Mycobacterium tuberculosis” | “Ação Integrada de Saúde”  “Ação Intersetorial”  “Ação Multissetorial”  “Ações Integradas de Saúde”  “Cooperação Intersetorial”  “Integração dos Serviços de Saúde”  “Intersetorialidade”  “Rede Intersetorial”  “Redes Intersetoriais”  “Acciones Integradas de Salud”  “Acción Integrada de Salud”  “Acción Intersectorial”  “Acción Multisectorial”  “Cooperación Intersectorial”  “Integración de los Servicios de Salud”  “Intersectorialidad”  “Red Intersectorial”  “Redes Intersectoriales” | Atenção Integral à Saúde  Atendimento Integral à Saúde  Estratégias Nacionais de Saúde Estratégias Sanitárias Nacionais  Saúde coletiva  Estrategias de Salud Nacionales  Atención Integral al Anciano Prestación Positiva de Servicios de Salud  Estrategias Nacionales de Salud Estrategias Sanitarias Nacionales | |
| **Construction** | | Tuberculose **OR** Tuberculosis  **OR** TB **OR**  “Pneumologia Sanitária” **OR**  “Infecção por Mycobacterium tuberculosis” **OR**  “Infección por Mycobacterium tuberculosis” | “Colaboração Intersetorial” **OR**  “Colaboración Intersectorial” **OR**  “Ação Integrada de Saúde” **OR**  “Ação Intersetorial” **OR**  “Ação Multissetorial” **OR**  “Ações Integradas de Saúde” **OR**  “Cooperação Intersetorial” **OR**  “Integração dos Serviços de Saúde” **OR**  “Intersetorialidade” **OR**  “Rede Intersetorial” **OR**  “Redes Intersetoriais” **OR**  “Acciones Integradas de Salud” **OR**  “Acción Integrada de Salud” **OR**  “Acción Intersectorial” **OR**  “Acción Multisectorial” **OR**  “Cooperación Intersectorial” **OR**  “Integración de los Servicios de Salud” **OR**  “Intersectorialidad” **OR**  “Red Intersectorial” **OR**  “Redes Intersectoriales” | “Assistência Integral à Saúde”” **OR**  “Programas Nacionais de Saúde” **OR**  “Estratégias de Saúde Nacionais” **OR**  “Gestão em saúde” **OR**  “Saúde Pública” **OR**  “Atención Integral de Salud” **OR**  “Programas Nacionales de Salud” **OR**  “Estrategias de Salud Nacionales” **OR**  “Gestión en Salud” **OR**  “Salud Pública” **OR**  “Atenção Integral à Saúde” **OR**  “Atendimento Integral à Saúde” **OR** “Estratégias Nacionais de Saúde” **OR** “Estratégias Sanitárias Nacionais” **OR**  “Estrategias de Salud Nacionales” **OR**  “Atención Integral al Anciano” **OR** “Prestación Positiva de Servicios de Salud” **OR**  “Estrategias Nacionales de Salud” **OR** “Estrategias Sanitarias Nacionales” | |
| **Use of the Brazilian Digital Library of Theses and Dissertations** ( **BDTD)** | | | | | **Records found** |
| **#1** | tuberculose OR “TB” OR “pneumologia sanitária” | | | | 12035 |
| **#2** | “colaboração intersetorial” OR “ação integrada de saúde” OR “ação intersetorial” OR “ação multissetorial” OR “ações integradas de saúde” OR “cooperação intersetorial” OR “integração dos serviços de saúde” OR “intersetorialidade” OR “rede intersetorial” OR “redes intersetoriais” | | | | 1628 |
| **#3** | “assistência integral à saúde” OR “programas nacionais de saúde” OR “estratégias de saúde nacionais” OR “gestão em saúde” OR “saúde pública” OR “atenção integral à saúde” OR “atendimento integral à saúde” OR “estratégias nacionais de saúde” OR “estratégias sanitárias nacionais” | | | | 29551 |
| **#1 #2 #3** | Tuberculose OR “TB” OR “Pneumologia Sanitária” AND “Colaboração Intersetorial” OR “Colaboración Intersectorial” OR “Ação Integrada de Saúde” OR “Ação Intersetorial” OR “Ação Multissetorial” OR “Ações Integradas de Saúde” OR “Cooperação Intersetorial” OR “Integração dos Serviços de Saúde” OR “Intersetorialidade” OR “Rede Intersetorial” OR “Redes Intersetoriais” AND “Assistência Integral à Saúde” OR “Programas Nacionais de Saúde” OR “Estratégias de Saúde Nacionais” OR “Gestão em saúde” OR “Saúde Pública” OR “Atenção Integral à Saúde” OR “Atendimento Integral à Saúde” OR “Estratégias Nacionais de Saúde” OR “Estratégias Sanitárias Nacionais” | | | | 0 |
| **#1 #2** | tuberculose OR “TB” OR “pneumologia sanitária” AND “colaboração intersetorial” OR “ação integrada de saúde” OR “ação intersetorial” OR “ação multissetorial” OR “ações integradas de saúde” OR “cooperação intersetorial” OR “integração dos serviços de saúde” OR “intersetorialidade” OR “rede intersetorial” OR “redes intersetoriais” | | | | 0 |
| **#1 #3** | Tuberculose OR “TB” OR “Pneumologia Sanitária” OR “Infecção por Mycobacterium tuberculosis” AND “Assistência Integral à Saúde” OR “Programas Nacionais de Saúde” OR “Estratégias de Saúde Nacionais” OR “Gestão em saúde” OR “Saúde Pública” OR “Atenção Integral à Saúde” OR “Atendimento Integral à Saúde” OR “Estratégias Nacionais de Saúde” OR “Estratégias Sanitárias Nacionais” | | | | 37 |

| **Objective** | | To map the approaches and results of intersectoral tuberculosis control actions at the global level, with a focus on intersectorality. | | | |
| --- | --- | --- | --- | --- | --- |
|  | | **P** | **C** | **C** | |
| **Extraction** | | Tuberculose | Intersetorialidade | Programas Nacionais de Controle da Tuberculose  Politica Nacional de Controle da Tuberculose  Ações de controle da Tuberculose  Saúde Pública | |
| **Conversion** | | Tuberculose  Tuberculosis | Colaboração Intersetorial  Colaboración Intersectorial | Assistência Integral à Saúde  Programas Nacionais de Saúde  Estratégias de Saúde Nacionais  Gestão em saúde  Saúde Pública  Atención Integral de Salud  Programas Nacionales de Salud  Estrategias de Salud Nacionales  Gestión en Salud  Salud Pública | |
| **Combination** | | TB  “Pneumologia Sanitária”  “Infecção por Mycobacterium tuberculosis”  “Infección por Mycobacterium tuberculosis” | “Ação Integrada de Saúde”  “Ação Intersetorial”  “Ação Multissetorial”  “Ações Integradas de Saúde”  “Cooperação Intersetorial”  “Integração dos Serviços de Saúde”  “Intersetorialidade”  “Rede Intersetorial”  “Redes Intersetoriais”  “Acciones Integradas de Salud”  “Acción Integrada de Salud”  “Acción Intersectorial”  “Acción Multisectorial”  “Cooperación Intersectorial”  “Integración de los Servicios de Salud”  “Intersectorialidad”  “Red Intersectorial”  “Redes Intersectoriales” | Atenção Integral à Saúde  Atendimento Integral à Saúde  Estratégias Nacionais de Saúde Estratégias Sanitárias Nacionais  Saúde coletiva  Estrategias de Salud Nacionales  Atención Integral al Anciano Prestación Positiva de Servicios de Salud  Estrategias Nacionales de Salud Estrategias Sanitarias Nacionales | |
| **Construction** | | Tuberculose **OR** Tuberculosis  **OR** TB **OR**  “Pneumologia Sanitária” **OR**  “Infecção por Mycobacterium tuberculosis” **OR**  “Infección por Mycobacterium tuberculosis” | “Colaboração Intersetorial” **OR**  “Colaboración Intersectorial” **OR**  “Ação Integrada de Saúde” **OR**  “Ação Intersetorial” **OR**  “Ação Multissetorial” **OR**  “Ações Integradas de Saúde” **OR**  “Cooperação Intersetorial” **OR**  “Integração dos Serviços de Saúde” **OR**  “Intersetorialidade” **OR**  “Rede Intersetorial” **OR**  “Redes Intersetoriais” **OR**  “Acciones Integradas de Salud” **OR**  “Acción Integrada de Salud” **OR**  “Acción Intersectorial” **OR**  “Acción Multisectorial” **OR**  “Cooperación Intersectorial” **OR**  “Integración de los Servicios de Salud” **OR**  “Intersectorialidad” **OR**  “Red Intersectorial” **OR**  “Redes Intersectoriales” | “Assistência Integral à Saúde”” **OR**  “Programas Nacionais de Saúde” **OR**  “Estratégias de Saúde Nacionais” **OR**  “Gestão em saúde” **OR**  “Saúde Pública” **OR**  “Atención Integral de Salud” **OR**  “Programas Nacionales de Salud” **OR**  “Estrategias de Salud Nacionales” **OR**  “Gestión en Salud” **OR**  “Salud Pública” **OR**  “Atenção Integral à Saúde” **OR**  “Atendimento Integral à Saúde” **OR** “Estratégias Nacionais de Saúde” **OR** “Estratégias Sanitárias Nacionais” **OR**  “Estrategias de Salud Nacionales” **OR**  “Atención Integral al Anciano” **OR** “Prestación Positiva de Servicios de Salud” **OR**  “Estrategias Nacionales de Salud” **OR** “Estrategias Sanitarias Nacionales” | |
| **Use in RCAAP** | | | | | **Records found** |
| **#1** | tuberculose OR pneumologia sanitária | | | | 4472 |
| **#2** | colaboração intersetorial OR intersetorialidade | | | | 17005 |
| **#3** | assistência integral à saúde OR programas nacionais de saúde OR estratégias de saúde nacionais | | | | 70 |
| **#1 #2 #3** | Tuberculose OR Pneumologia Sanitária AND Colaboração Intersetorial OR Intersetorialidade AND assistência integral à saúde OR programas nacionais de saúde OR estratégias de saúde nacionais | | | | 0 |
| **#1 #2** | Tuberculose OR Pneumologia Sanitária AND Colaboração Intersetorial OR Intersetorialidade | | | | 28 |
| **#1 #3** | Tuberculose OR Pneumologia Sanitária AND assistência integral à saúde OR programas nacionais de saúde OR estratégias de saúde nacionais | | | | 2 |

| **Objective** | | To map the approaches and results of intersectoral tuberculosis control actions at the global level, with a focus on intersectorality. | | | |
| --- | --- | --- | --- | --- | --- |
|  | | **P** | **C** | **C** | |
| **Extraction** | | Tuberculosis | “Intersectoral Collaboration” | “Comprehensive Health Care”  “National Health Programs”  “Population Health Management”  “Public Health” | |
| **Conversion** | | Tuberculosis | “Intersectoral Collaboration” | “Comprehensive Health Care”  “National Health Programs”  “Population Health Management”  “Public Health” | |
| **Combination** | | Tuberculoses  “Koch's Disease”  “Koch Disease”  “Mycobacterium tuberculosis Infection”  “Infection, Mycobacterium tuberculosis”  “Infections, Mycobacterium tuberculosis”  “Mycobacterium tuberculosis Infections” | “Collaboration, Intersectoral”  “Collaborations, Intersectoral”  “Intersectoral Collaborations”  “Intersectoral Cooperation”  “Cooperation, Intersectoral” | “Health Care, Comprehensive”  “Comprehensive Healthcare”  “Healthcare, Comprehensive”  “Health Program, National”  “Health Programs, National”  “National Health Program”  “Program, National Health”  “Programs, National Health”  “Health Services, National”  “Health Service, National”  “National Health Service”  “Service, National Health”  “Services, National Health”  “National Health Services”  “Health Management, Population”  “Management, Population Health”  “Community Health” | |
| **Construction** | | Tuberculosis **OR** Tuberculoses **OR** “Kochs Disease” **OR** “Koch's Disease” **OR** “Koch Disease” **OR** “Mycobacterium tuberculosis Infection” **OR** “Infection, Mycobacterium tuberculosis” **OR** “Infections, Mycobacterium tuberculosis” **OR**  “Mycobacterium tuberculosis Infections” | “Intersectoral Collaboration” **OR** “Collaborations, Intersectoral” **OR** “Intersectoral Collaborations” **OR** “Intersectoral Cooperation” **OR**  “Cooperation, Intersectoral” | “Comprehensive Health Care” **OR** “National Health Programs” **OR** “Population Health Management” **OR** “Health Care, Comprehensive” **OR**  “Comprehensive Healthcare” **OR**  “Healthcare, Comprehensive” **OR**  “Health Program, National” **OR**  “Health Programs, National” **OR**  “National Health Program” **OR**  “Program, National Health” **OR**  “Programs, National Health” **OR**  “Health Services, National” **OR**  “Health Service, National” **OR**  “National Health Service” **OR**  “Service, National Health” **OR**  “Services, National Health” **OR**  “National Health Services” **OR** “Public Health” **OR** “Community Health” **OR** “Health Management, Population” **OR** “Management, Population Health” | |
| ***Use in WHO Global Research** | | | | | **Records found** |
| **#1** | tuberculosis | | | | 133 |
| **#2** | Intersectoral | | | | 05 |
| **#3** | Comprehensive Health Care | | | | 01 |
| **#1 #2 #3** | Tuberculosis **AND** Intersectoral **AND** Comprehensive Health Care | | | | 00 |
| **#1 #2** | Tuberculosis **AND** Intersectoral | | | | 00 |
| **#1 #3** | Tuberculosis **AND** “Comprehensive Health Care | | | | 00 |

*The 133 **WHO Global Research** #1 files were considered for the review.

Data collection ended on September 26, 2024.
