# Supplementary figures and images for: Approaches and results of intersectoral actions for tuberculosis control in the world: A scoping review
Source: PLoS One. 2025 Jun 26;20(6):e0326784. doi: 10.1371/journal.pone.0326784 (PMC12200668; doi:10.1371/journal.pone.0326784)

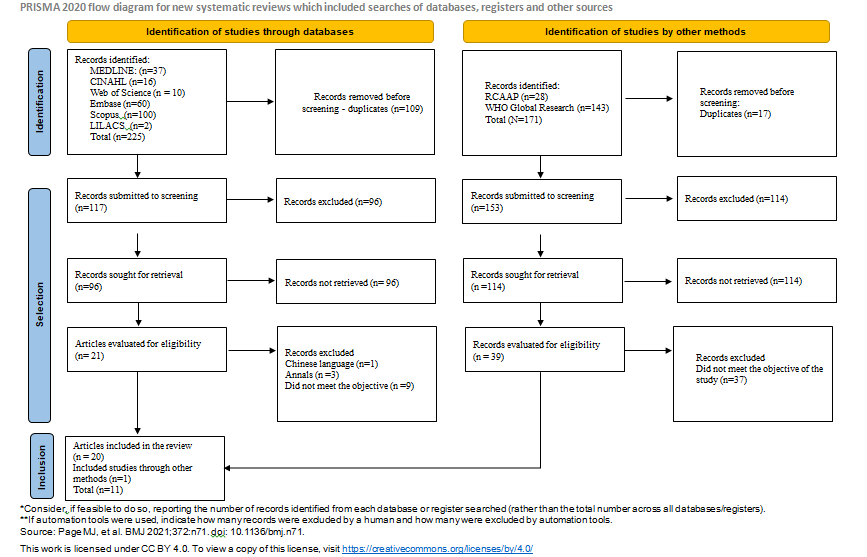

Supplement: S3 Fig — Of the study selection process. (TIF) [file pone.0326784.s003.tif]

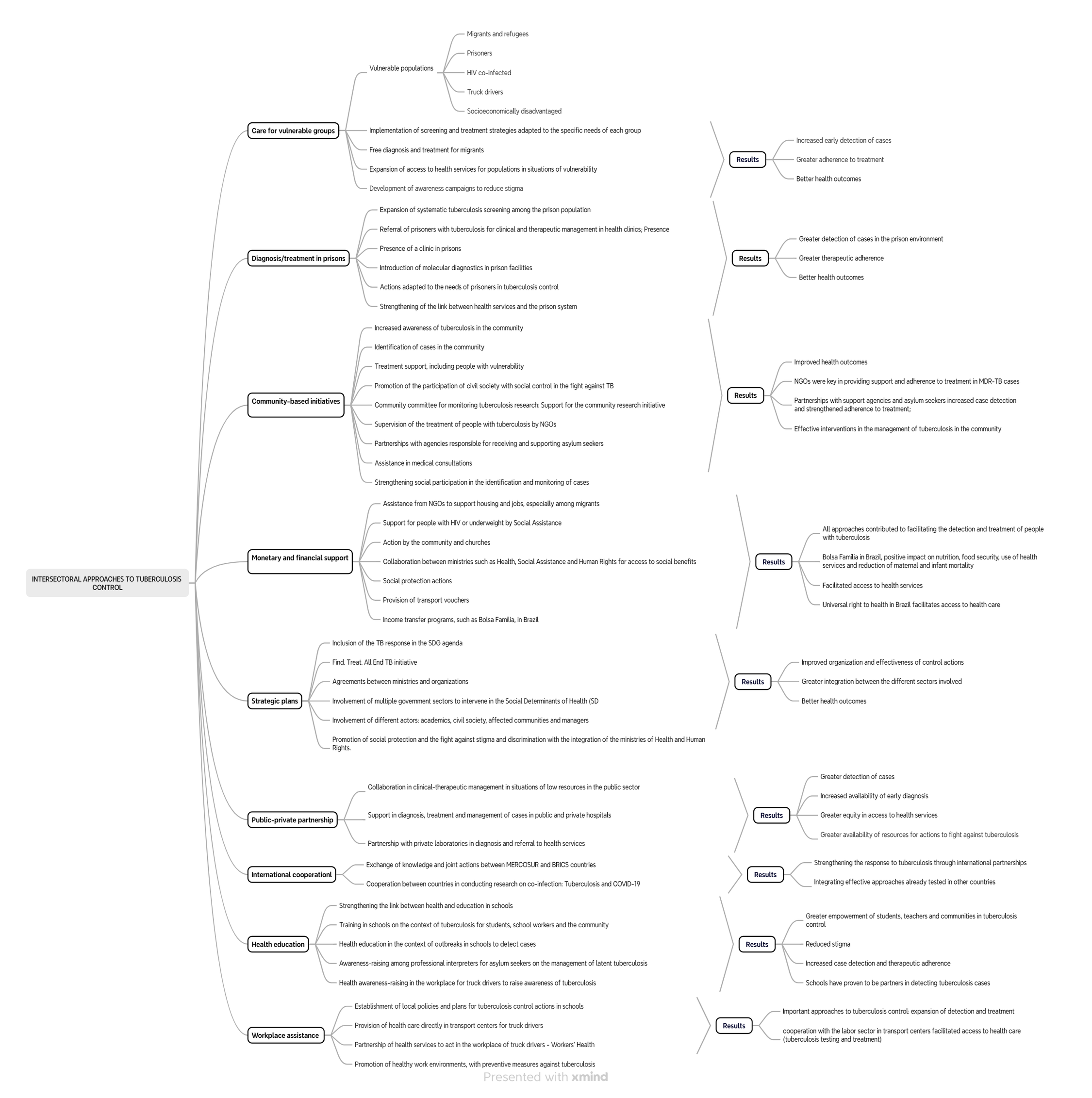

Supplement: S4 Fig — The figure presents the visual synthesis of the approaches and results identified in the scoping review. It structures and systematizes the information to improve the understanding of the mapped intersectoral approaches and results. (TIF) [file pone.0326784.s004.tif]
