# Supplementary material for: Approaches and results of intersectoral actions for tuberculosis control in the world: A scoping review
Source: PLoS One. 2025 Jun 26;20(6):e0326784. doi: 10.1371/journal.pone.0326784 (PMC12200668; doi:10.1371/journal.pone.0326784)
Supplement: Attachment — Submitted filename: Response to Reviewers.docx [file pone.0326784.s006.docx]

Dear Editor, we are very grateful for your valuable contributions, which were accepted. They were very important to make the manuscript more robust and suitable for the journal. The response will be made according to the notes, and if you find any other details that need improvement, please let us know, and we will be happy to improve them.

1. A review was carried out on the adequacy of the manuscript to the PLOS ONE style according to the sources indicated, as noted in the file.

2. The funding source was corrected in the cover letter and in the additional information section regarding financial disclosure.

3. The support files used for this review are all attached to the submission to the journal, as noted in the attachments Support file - Spreadsheets for storing data; Support Files - Search Strategies and PRISMA-ScR-Fillable Checklist and we are sending a data sharing plan to the journal signed by the authors.

4. The legends for the supplementary information files were included at the end of the Lines manuscript 489 to 499 and all citations were reviewed to properly match the PLOS ONE style

- Response to additional comments from the Academic Editor

1. The definition of Intersectorality was inserted: Lines: 69-74.

2. **Is population tuberculosis?** No, according to JBI recommendations, the mnemonic combination of PCC is used, which can correspond to Problem or population, Concept and Context. Therefore, the term “Problem” was used and not “Population” in the respective study; however, if a replacement for the term “Population” is necessary, we can do so.

3. Regarding the concept, in this study we proposed to identify approaches and results of intersectoral actions in the studies that were found. Therefore, we did not limit ourselves to a precise search for the sectors, although it was clear that the sectors of social assistance, community and family support, collaborations between countries, public-private relations, schools and the prison system are acting in an intersectoral manner in tuberculosis care. According to this specification, if we break down the concept by pointing out the possible sectors, more elements may appear, but not necessarily in a collaborative manner, which would be at odds with the primary objective of this study.

4. The choice of OSF was made due to its broader scope and its frequent use by researchers in Brazil and abroad, including for review purposes. Although we are not very familiar with the Prospero platform, in future studies we propose to review this issue and get to know it more deeply. We appreciate the suggestion.

5.The translation mistake in line 128 regarding intersectorality was corrected: Line: 108.

6. Although it was not mentioned due to the scarcity of studies on the programs, tuberculosis control in all countries is directly managed by National Tuberculosis Control Programs, which follow a specific hierarchy as recommended by The End-TB strategy. These programs are decentralized, and the World Health Organization (WHO) has assigned National Tuberculosis Control Programs the responsibility of implementing tuberculosis control actions in the countries. Although some studies do not directly mention the programs, they indicate that the actions are coordinated by local health. For this reason, the study included these studies that portray intersectoral action within these structures with a focus on their approaches and results; we did not intend to analyze or evaluate such actions in each nationality.

7. The year threshold is shown in the line: 162.

8. Studies in all languages were not included, as stated in the Lines: 159-160.

9. All search data from the supplementary file “Search strategies” are available according to their adaptations and number of studies found 488-500.

10. Correction made in Table 3 in the study by “Yuen et al”, the term “were found” was removed

11. Table 3 was reformulated with the creation of the results column, to separate the approaches from the results, as requested: Line: 207.

12. Adjustments in the conclusion were made: Lines: 456-486.

Reply to Reviewer 1.

1. Adjustments in the results section were made, Lines: 207-216.
2. Regarding the annals and editorials, they are short and superficial texts. Regarding the books, it would not be possible to include updated books on the subject. The decision to include reviews, manuals, technical and government publications in the review, while editorials, experience reports, annals and books were excluded, is based on the need to ensure the quality, reliability and applicability of the data analyzed. Systematic reviews, manuals and institutional publications generally follow strict methodological criteria and are supported by scientific evidence or normative guidelines, which contributes to greater robustness in the findings. It is recognized that some excluded categories could contain relevant information, but it was decided to focus on sources that present a higher degree of methodological standardization and a lower degree of subjectivity. The exclusion of editorials and books is due to the fact that these materials often do not follow the same peer review criteria and may contain more opinionated interpretations. Regarding the possibility of reviews including editorials and books in their analyses, it is worth noting that the selection of reviews took into account their search and inclusion methodology, in order to minimize bias in the interpretation of the results. Thus, the exclusion criteria were established to improve the consistency and validity of the review performed.
3. The term “sample” was replaced in Table 2. Line: 195.
4. Adjustment was made; the results remain in the table, but the conceptual structure (Fig 3) presents the data with short sentences: Lines: 205-216.
5. The conceptual structure was created Fig 2, Line: 212.
6. The discussion and conclusion were reduced as suggested: Lines: 218-486, if it is necessary to reduce it even further, please do not hesitate to let us know. We did not reduce it further because we were concerned about leaving out important results.

-Reply to Reviewer 2

1. We appreciate your comments on the study. The process of retrieving studies was carried out in a detailed manner, aiming to achieve the best results that met the objective of the study, as verified in the support file of the search strategies. As an example, in the gray literature, especially in the “WHO Global Research” database, we carefully examined 133 publications. When these publications mentioned multisectoral/intersectoral actions in some location, we always consulted the original sources. However, we found that most presented more intersectoral strategies than concrete actions effectively carried out (approaches and their results), which is at odds with our analysis objective. For this reason, these publications were not included in our analysis. It is important to emphasize that the objective was to evaluate the tuberculosis control actions that were carried out and their results; the case studies found reflected more strategies/recommendations.

2. It is important to clarify that the primary intention of our study was to focus on intersectoral collaboration for tuberculosis control. The identified sectors emerged throughout the research process and were not previously anticipated. Even if the sectors were included, the results could be expanded, but not necessarily in a collaborative manner, which would be at odds with the primary objective of this study.

3. Changes were made in Table 3 to separate the results, Line: 207.

4. Changes were made in the conclusion, lines: 456-486.

5. Responses to minor issues:

5.a. Revised use of terminology and translation

5.b. Corrected, Lines: 61-63.

Note: We are resending the PRISMA checklist and supporting files S1 Search Strategies, S2 Spreadsheets for storing data, with the term “PubMed” replaced by “Medline”, as Medline is the more precise term when referring to the database.
