# Supplementary material for: Approaches and results of intersectoral actions for tuberculosis control in the world: A scoping review
Source: PLoS One. 2025 Jun 26;20(6):e0326784. doi: 10.1371/journal.pone.0326784 (PMC12200668; doi:10.1371/journal.pone.0326784)
Supplement: Attachment — Submitted filename: Response_to_Reviewers_auresp_2.docx [file pone.0326784.s007.docx]

Dear Publisher,

Reply to Reviewer 2.

We would like to thank you very much for your valuable contributions, all of which have been accepted. They were of great importance in making the manuscript more robust and suitable for the journal. The reply will be drafted according to the observations made. If there are any other details that need improvement, please let us know; we will be happy to improve them.

1. Redrafted the “Final Considerations”. Lines: 452-490.

2. Adjustments made to the study’s limitations. Lines: 474-485.

3. Adjustments made to the terms “Eliminate” and “Elimination”. Lines: 19; 58; 63; 65; 256. All excluded.

Thank you!
